# Multiplicity of Infection and Disease Severity in *Plasmodium vivax*

**DOI:** 10.1371/journal.pntd.0004355

**Published:** 2016-01-11

**Authors:** M. Andreína Pacheco, Mary Lopez-Perez, Andrés F. Vallejo, Sócrates Herrera, Myriam Arévalo-Herrera, Ananias A. Escalante

**Affiliations:** 1 Institute for Genomics and Evolutionary Medicine (igem), Temple University, Philadelphia, Pennsylvania, United States of America; 2 Caucaseco Scientific Research Center and Malaria Vaccine and Drug Development Center, Cali, Colombia; 3 Faculty of Health, Universidad del Valle, Cali, Colombia; Johns Hopkins Bloomberg School of Public Health, UNITED STATES

## Abstract

**Background:**

Multiplicity of infection (MOI) refers to the average number of distinct parasite genotypes concurrently infecting a patient. Although several studies have reported on MOI and the frequency of multiclonal infections in *Plasmodium falciparum*, there is limited data on *Plasmodium vivax*. Here, MOI and the frequency of multiclonal infections were studied in areas from South America where *P*. *vivax* and *P*. *falciparum* can be compared.

**Methodology/Principal Findings:**

As part of a passive surveillance study, 1,328 positive malaria patients were recruited between 2011 and 2013 in low transmission areas from Colombia. Of those, there were only 38 *P*. *vivax* and 24 *P*. *falciparum* clinically complicated cases scattered throughout the time of the study. Samples from uncomplicated cases were matched in time and location with the complicated cases in order to compare the circulating genotypes for these two categories. A total of 92 *P*. *vivax* and 57 *P*. *falciparum* uncomplicated cases were randomly subsampled. All samples were genotyped by using neutral microsatellites. *Plasmodium vivax* showed more multiclonal infections (47.7%) than *P*. *falciparum* (14.8%). Population genetics and haplotype network analyses did not detect differences in the circulating genotypes between complicated and uncomplicated cases in each parasite. However, a Fisher exact test yielded a significant association between having multiclonal *P*. *vivax* infections and complicated malaria. No association was found for *P*. *falciparum* infections.

**Conclusion:**

The association between multiclonal infections and disease severity in *P*. *vivax* is consistent with previous observations made in rodent malaria. The contrasting pattern between *P*. *vivax* and *P*. *falciparum* could be explained, at least in part, by the fact that *P*. *vivax* infections have lineages that were more distantly related among them than in the case of the *P*. *falciparum* multiclonal infections. Future research should address the possible role that acquired immunity and exposure may have on multiclonal infections and their association with disease severity.

## Author Summary

Previous studies on rodent malarias and mathematical models have postulated a link between multiclonal infections and disease severity. This association has been tested in *Plasmodium falciparum* mostly in Africa with limited information on *P*. *vivax*. Furthermore, there is a paucity of information from areas with low transmission. Here, we used samples available from a passive surveillance carried out in Colombia, South America. We found an association between multiclonal infections and disease severity in *P*. *vivax* but not in *P*. *falciparum*. Although the number of complicated malaria cases is low, the contrasting pattern between these two species emphasizes their epidemiological differences. We discuss how this pattern could be the result of a higher divergence among the *P*. *vivax* lineages co-infecting a patient. We hypothesize that low levels of acquired immunity may play a role in the association between multiclonal infections and disease severity.

## Introduction

A common observation in many malaria endemic areas is that there are patients concurrently infected by more than one distinct parasite genotype. These infections are usually referred to as multiclonal infections. In addition to the frequency of multiclonal infections, molecular epidemiologists estimate the average number of lineages per infected individual or multiplicity of infection (MOI). Together, MOI and the frequency of multiclonal infections are measurements that relate to transmission intensity [1, 2]. Ecologically, multiclonal infections could be the result of two different processes, the co-transmission of different parasite variants (co-infections) or the overlap of genetic variants due to infectious contacts before the primary infection is resolved (superinfections) [1, 2]. Distinguishing between these processes is particularly laborious in field settings.

Beyond their expected association with transmission, multiclonal infections may indicate complex interactions between genetically distinct parasite lineages and their host [2, 3]. Broadly defined as intra-host dynamics, these interactions have been the subject of several theoretical and experimental studies. These processes are considered related to disease severity and the fixation of mutations associated with drug resistance [2–7]. In particular, mathematical models and data from experimental infections in rodent malaria observed that, as result of competition among genetically distinct lineages, multiclonal infections should be more virulent than single infections. Furthermore, they could lead to an increase in virulence at the population level because natural selection will favor parasites that, by replicating more, outcompete less virulent variants [5]. This hypothesis, however, had limited empirical support from field epidemiologic investigations [3, 8].

The estimation of MOI and the frequency of multiclonal infections as part of epidemiological studies have several technical limitations that hamper our ability to compare field observations with predictions made in terms of disease severity from laboratory models (S1 Table). First, MOI is usually reported as a single measurement on a locus or set of loci. Most studies actually ignore whether there are undetectable genetic differences that are phenotypically relevant. Second, there is no standardization on the loci used; thus, comparisons across studies are intrinsically difficult [8–13]. The matter is further complicated by the fact that many MOI studies have been carried out using the fragment size polymorphisms of genes encoding antigens such as *msp-2* in *Plasmodium falciparum* and *msp3α* in *Plasmodium vivax*. Variation at these loci may be hard to interpret since multiple insertion-deletion mutations, recombination events, and/or convergence due to selection could generate alleles that differ at the sequence level but have the same fragment size or restriction fragment length polymorphism pattern [9, 14, 15]. Finally, many studies lack suitable controls in terms of potential differences in parasite genotypes circulating among the group of patients compared. Such comparison is important since observations from a rodent malaria model suggest that genotypes may differ in their competitive capabilities [5, 16, 17]. Indeed, results from these experimental infections support the hypothesis that different genotypes may lead to different outcomes. On top of these technical problems, the role of immunity and the actual temporal dynamics of how different genotypes interact within the host are factors that make any association between MOI and disease severity hard to detect in field studies [3, 16].

Regardless of these challenges, the hypothesized links between multiclonal infections and clinical outcomes have driven several molecular epidemiologic investigations [3, 18]. Not surprisingly, the data emerging from field studies on *P*. *falciparum* are contradictory [3]. Some studies report associations between MOI and clinical endpoints [10, 19–21] and others fail to find any or find that single clonal infections are actually associated with severe disease [12, 22–27]. Regretfully, there are only a handful of studies that include *P*. *vivax* or that are carried out in low transmission settings (see S1 Table).

Here, the relationships between the frequency of multiclonal infections and MOI with complicated malaria were explored in areas with seasonal transmission in Colombia, South America. These settings offer three advantages. First, finding single infections is possible so they can be compared with multiple infections in the context of complicated/uncomplicated malaria cases. Second, *P*. *vivax* can be compared with *P*. *falciparum* in the same population. Finally, exposure to malaria is lower than in hyper-endemic areas in Africa, Southeast Asia, and Oceania/Pacific so a lower impact of acquired immunity is expected. A problem in these low transmission areas, however, is that the number of reported complicated malaria cases is limited so studies need to be carried out for longer periods of time [28, 29]. In this investigation, the complexities of infections were compared in *P*. *vivax* and *P*. *falciparum* cases by using multiple species-specific microsatellite loci. These loci offer the advantage of being neutral if they are not physically linked to a gene under selection (e.g. mutations conferring drug resistance or antigens). Furthermore, a multi-locus approach that incorporates fast evolving microsatellite loci can detect multiple infections even when the lineages co-infecting the patient are highly related, a phenomenon expected in low transmission areas [11, 13].

Although *P*. *vivax* may result in slightly more complex infections than *P*. *falciparum* (higher MOI), no noticeable differences were found in terms of the average MOI between complicated and uncomplicated cases in these two parasites. No detectable differences were found in *P*. *vivax* or *P*. *falciparum* in terms of specific multilocus genotypes infecting complicated and uncomplicated cases. However, we found that multiclonal infections were associated with complicated malaria in *P*. *vivax* but not in *P*. *falciparum*.

## Materials and Methods

### Ethics statement and study design

A passive surveillance study was conducted between 2011 and 2013 in four malaria outpatient clinics located in areas with distinct transmission intensities and parasite distribution [29]. A total of 1,328 symptomatic volunteers were passively recruited when visiting the health posts for malaria diagnosis. Patients with malaria infection as determined by microscopic examination of Giemsa stained thick blood smears (TBS) received oral and written explanations about the study and, after free willingness to participate, were requested to sign an informed consent (IC) form previously approved by the Institutional Review Board (IRB) affiliated to the Malaria Vaccine and Drug Development Center (MVDC, Cali-Colombia). IC from each adult individual or informed assent (IA) from the parents or guardians of children <18 years of age was obtained. Individuals between seven and 17 years of age were asked to sign an additional IA. A trained physician of the study staff completed a standard clinical evaluation and a physical examination in all malaria symptomatic subjects. All individuals were treated by the local health provider as soon as the blood sample was drawn, using the national antimalarial therapy protocol of the Colombian Ministry of Health and Social Protection (MoH) [29]. Each individual received a unique code number to simplify data collection and identification. Out of the 1,328 patients, 38 *P*. *vivax* and 24 *P*. *falciparum* cases were classified as clinically complicated following the criteria listed in Table 1. These complicated cases were scattered throughout the time of the study. In order to control for temporal fluctuations of malarial genotypes, complicated cases were compared with a random subsample of uncomplicated cases that were diagnosed in a time window of up to 8 days around each complicated case and in the same locality. As a result, 92 *P*. *vivax* and 57 *P*. *falciparum* uncomplicated cases were randomly subsampled and genotyped as described below.

**Table 1:** Complicated malaria criteria used in this study.

Criteria	Description [32, 33]	*P*. *vivax* n = 38	*P*. *falciparum* n = 24
**Only one criteria:**			
Hepatic dysfunction	Total bilirubin > 3 mg/dL or alanine aminotransferase > 120 U/L.	11 (28.9%)	6 (25%)
Renal dysfunction	Serum creatinine > 1.5 mg/dL or blood urea nitrogen (BUN) > 40 mg/dL.	7 (18.4%)	3 (12.5%)
Prostration	Generalized weakness where patient is unable to walk or sit up without assistance.	4 (10.5%)	6 (25%)
Hemoglobinuria	Macroscopic and positive urine dipstick, in absence of microscopic haematuria.	7 (18.4%)	0
Respiratory distress	Presence of alar flaring, chest recession or abnormal deep or acidotic breathing.	1 (2.6%)	0
Hyperparasitemia	>50,000 asexual parasites/μL.	2 (5.3%)	0
Abnormal spontaneous bleeding	Spontaneous bleeding in the presence of laboratory evidence of DIC.	0	1 (4.2%)
Severe anemia	Hemoglobin < 7g/dL.	2 (5.3%)	1 (4.2%)
Severe thrombocytopenia	< 20,000 platelets/μL.	1 (2.6%)	1 (4.2%)
**More than one criteria:**			
Hepatic dysfunction plus	Severe thrombocytopenia	1 (2.6%)	0
	Prostration	0	2 (8.3%)
	Hyperparasitemia	0	1 (4.2%)
Renal dysfunction plus	Prostration	1 (2.6%)	0
	Hyperparasitemia	0	1 (4.2%)
Hyperparasitemia plus	Respiratory distress	0	1 (4.2%)
	Hemoglobinuria	1 (2.6%)	0
	Severe thrombocytopenia	0	1 (4.2%)

### Study sites

Four localities in Colombia were selected due to their high prevalence of malaria but different average annual parasite incidence (API) between 2011 and 2013: Tierralta (Department of Córdoba; API ~6.7) in the northern area, Quibdó (Department of Chocó API ~25), Buenaventura (Department of Valle del Cauca; API ~1.9) and Tumaco (Department of Nariño; API ~10.3) in the southeast area of the Pacific Coast. *Plasmodium vivax* and *P*. *falciparum* are both transmitted in these regions in different proportions with an unstable endemic pattern, displaying differences in the relative importance of both parasites. As in other areas of Latin America [30], the incidence of *P*. *falciparum* has been declining in recent years across the sampled localities whereas *P*. *vivax* has shown to be a more resilient parasite. In Tierralta (~90,000 inhabitants), 44.4% of the population lives in the rural areas. Most inhabitants are mestizos and a small Amerindian indigenous community of Emberá Katío. In this region, the predominant malaria parasite species is *P*. *vivax* (~85%). Quibdó (~100,000 inhabitants), located on the Pacific Coast of Colombia close to the border with Panamá, has a population mainly consisting of Afro-descendants and Afro-Amerindians. Most of the malaria cases are caused by *P*. *falciparum* (~70%). In Buenaventura (350,000 inhabitants), most of inhabitants are Afro-descendants and mestizos and more than 90% of the population lives in the urban area with malaria mostly due to *P*. *vivax* (~75%). Tumaco (~160,000 inhabitants) is situated close to the border with Ecuador with a population predominantly Afro-descendants with an Amerindian indigenous community of Awá the predominant malaria parasite species in this region is *P*. *falciparum* (~79%)[29].

### Clinical assessment

Approximately 100 μL of whole blood were collected by finger-prick. Malaria diagnosis at enrollment was performed by Giemsa stained TBS and examined under oil immersion by an expert microscopist and the parasitemia was confirmed by a second experienced reader [29]. Parasite density was counted after reviewing at least 200 leukocytes. Total parasite load was expressed as the number of parasites/μL using the actual leukocytes counts for each patient. Then, DNA was extracted using the PureLink Genomic DNA kit (Invitrogen, USA) and Real time PCR (RT-qPCR) was performed retrospectively as described elsewhere [31] to confirm the parasite species. Standard *P*. *falciparum* and *P*. *vivax* DNA positive and negative controls were used in each batch of tests, including extraction of both negative and inhibition control. A sample was considered negative if there was no increase in the fluorescent signal after a minimum of 40 cycles.

### Complicated (CMC) and uncomplicated malaria cases

Regardless of the malaria parasite species, patients were classified as complicated malaria according to the clinical and laboratory criteria of the WHO and the Colombian Ministry of Health and Social Protection guidelines (Table 1)[32,33]. Uncomplicated malaria was defined as a clinical malaria case (symptoms including fever >38°C, headache, chills and/or malaise and a positive TBS) without severity criteria. Clinical and parasitological findings have been reported [29].

### Microsatellite (STRs) genotyping

Genomic DNAs were used for microsatellites analyses. Those samples with low parasitemia were amplified by whole genome amplification using the REPLI-g Mini Kit (Qiagen Inc, CA, USA). Genotyping was performed using fluorescently labeled PCR primers for a set of nine standardized microsatellite loci for *P*. *vivax* and nine for *P*. *falciparum* out of an extensive pool of loci that have been explored [11]. In the case of *P*. *vivax* the following loci were included in the analyses: MS2, MS5, MS6, MS15 [34] and 14.185, 8.332, 2.21, 10.29, and 8.332 [35]. Loci POLYa, TAA60, ARA2, Pfg377, TAA109, TAA81, TAA42-3, TA40, and PfPK2 were amplified for *P*. *falciparum* [36]. Fluorescently labeled PCR products were separated on an Applied Biosystems 3730 capillary sequencer and scored using GeneMarker v2.6.3 (SoftGenetics LLC). After the microsatellite pattern was identified across samples, we scored all the alleles at a given locus if minor peaks were more than one-third the height of the predominant peak. The finding of one or more additional alleles at any locus was interpreted as a multiple infection with two or more genetically distinct clones in the same isolate (transmitted by one or several mosquitoes). Single infections were those with only one allele per locus at all the genotyped loci; this method has been widely used [11,34–36]. Missing data (no amplifications) were reported by locus but not considered for defining multilocus genotypes.

### Population genetic analysis

Suit of approaches was used to test whether there were differences in the circulating genotypes in complicated and uncomplicated malaria cases by exploring how microsatellite haplotypes clustered. We used the Haplotype Analysis software v1.04 [37] on all the multi-locus genotypes that we could unambiguously identify. Thus, a limitation in these analyses is that complex infections with differences at more than two loci were not included because the haploid genotypes could not be inferred. In particular, we estimated the number of different sampled multilocus genotypes (SMG), number of unique genotypes (G), number of private genotypes (PG), and the Nei’s index of genetic diversity (He) estimated without bias [38]. The *He* was defined as *He* = [*n*/(*n* − 1)][$1-\sum_{i=1}^{L}p_{i}^{2}$], where *n* is the number of isolates analyzed and *p*_*i*_ is the frequency of the *i*-th allele (*i* = 1, …, L) in the population. *He* gives the average probability that a pair of alleles randomly selected from the population is different. Then, a Bayesian model-based clustering algorithm was used as implemented in the Structure v2.3.4 software [39]. This software uses a Bayesian clustering approach to assign isolates to *K* populations or clusters characterized by a set of allele frequencies at each locus. This approach allows for the identification of groups or populations of parasites that could separate the group of complicated and uncomplicated malaria. We evaluated the observed genetic diversity at different K values (K = 2 to 10 for *P*. *falciparum* and K = 2 to 30 for *P*. *vivax*) and each K value was run independently 10 times with a burn-in period of 10,000 iterations followed by 10,000 iterations. For this analysis, we used a set of eight out of the nine microsatellites for both parasites (without MS2 and PfPK2) in order to include as many samples as possible. The admixture model was used in all the analyses that allow for the presence of individuals with ancestry in two or more of the K populations [39]. We used Structure Harvester v0.6.94 to compute Delta K values from Structure [40]. The program CLUMPP (Cluster Matching and Permutation Program) was used to facilitate the interpretation of population-genetic clustering results [41], and then, *distruct* v1.1 was used to graphically display the clustering results [42]. The posterior probability for each number of populations or clusters (*K*) is computed and the *K* value that better explains the genetic data is an estimate of the number of circulating clusters or populations circulating.

Finally, population genetic analyses were complemented by inferring the haplotype genealogies found in complicated and uncomplicated malaria cases for each *Plasmodium* species. Those genealogies were inferred for eighth microsatellites by using the Global Optimal eBURST algorithm [43], as implemented in PHYLOViZ [44]. Using an extension of the goeBURST rules up to *n* locus variants level (***n*LV**, where ***n*** equals to the number of loci in our dataset: eight), a Minimum Spanning Tree-like structure was drawn to cluster the 100 sequence types (STs) for *P*. *vivax* and 18 for *P*. *falciparum* (including uncomplicated and complicated malaria cases) into a clonal complex (CC) based on their multilocus genotypes (a total of 130 patients infected with *P*. *vivax* and 81 patients with *P*. *falciparum*, many sharing the same sequence types).

### Statistical analysis

A Fisher exact test was performed for 130 *P*. *vivax* and 81 *P*. *falciparum* samples subdivided into uncomplicated and uncomplicated malaria cases (Table 1) to test the hypothesis that the frequency of multiclonal infections differs between complicated and uncomplicated malaria cases. Multiclonal infections were defined as those having more than one allele in at least one locus out of the nine loci genotyped. A single infection is one with only one allele per locus at all the genotyped loci.

## Results

There were few cases of complicated malaria in these areas with low transmission [29]. A total of 38 *P*. *vivax* and 24 *P*. *falciparum* cases were classified as clinically complicated following the criteria listed in Table 1. Uncomplicated cases were sub-sampled to create a control group that matched the complicate malaria cases (CMC) in terms of location and the time when the case was diagnosed. The age, gender, ethnic composition, average MOI (and range) and the percentage of multiplicity of infection of the complicated and uncomplicated malaria cases are reported in Table 2. No noticeable demographic differences were observed between the complicated and uncomplicated malaria groups (Table 2) and no association between gender and complicated and uncomplicated malaria cases was found (Table 3).

**Table 2:** Demographic data, average MOI (range) and percentage of multiclonal infection observed in *P*. *vivax* and *P*. *falciparum* samples.

	***P*. *vivax***		
	**Controls (N = 92)**	**CMC (N = 38)**	**Total (N = 130)**
**Age (years)**			
Range	3–68	4–74	3–74
Median	18.5	19.5	19
Average (±SD)	23.52±16.11	28.52±20.36	24.98±17.53
**Gender (%)**			
Male	68 (73.9)	24 (63.2)	92 (70.8)
Female	24 (26.1)	14 (36.8)	38 (29.2)
**Ethnicity (%)**			
Afro-descendant	36 (39.1)	17 (44.7)	53 (40.8)
Mestizo	44 (47.8)	18 (47.4)	62 (47.7)
Indigenous	8 (8.7)	2 (5.3)	10 (7.7)
White	4 (4.4)	1 (2.6)	5 (3.8)
**Average MOI (Range)**^**a**^	1.39 (1–2)	1.76 (1–3)	1.5 (1–3)
**Average MOI (SD)**^**b**^	1.40±0.49	1.68±0.66	1.48±0.56
**Multiclonal infection (%)**	39.1	68.4	47.7
	***P*. *falciparum***		
	**Controls (N = 57)**	**CMC (N = 24)**	**Total (N = 81)**
**Age (years)**			
Range	3–71	1–62	1–71
Median	22	19.5	20
Average (±SD)	25.98±15.31	23.67±14.76	25.25±15.18
**Gender (%)**			
Male	31 (54.4)	14 (58.3)	45 (55.5)
Female	26 (45.6)	10 (41.7)	36 (44.4)
**Ethnicity (%)**			
Afro-descendant	48 (84.2)	18 (75)	66 (81.5)
Mestizo	5 (8.8)	5 (20.8)	10 (12.4)
Indigenous	1 (1.7)	0	1 (1.2)
White	3 (5.3)	1 (4.2)	4 (4.9)
**Average MOI (Range)**^**a**^	1.16 (1–2)	1.13 (1–2)	1.15 (1–2)
**Average MOI (SD)**^**b**^	1.04±0.19	1.0±0.0	1.03±0.16
**Multiclonal infection (%)**	15.8	12.5	14.8

MOI estimated using: ^**a**^ all loci and ^**b**^ the locus with the highest number of alleles.

**Table 3:** Fisher exact test results.

	Species	*P*. *vivax*				*P*. *falciparum*		
**Gender**	**n = 130**	**Control**	**CMC**	**Total**	**n = 81**	**Control**	**CMC**	**Total**
	**Male**	68	24	92		31	14	45
	**Female**	24	14	38		26	10	36
	**Total**	92	38	130		57	24	81
	***p* value**	0.289				0.8097		
**Multiclonal Infections**	**n = 130**				**n = 81**			
	**Multiclonal**	36	26	62		9	3	12
	**Single**	56	12	68		48	21	69
	**Total**	92	38	130		57	24	81
	***p* value**	0.0035*				1.0		
	**n = 419**				**N = 279**			
	**Multiclonal**	187	26	213		56	3	59
	**Single**	194	12	206		199	21	220
	**Total**	381	38	419		255	24	279
	***p* value**	0.0268*				0.432		

Fisher tests were performed on *P*. *vivax* and *P*. *falciparum* samples for: (1) association between gender and complicated and uncomplicated malaria cases and (2) association between the frequency of multiclonal infections and complicated malaria cases (CMC). The *p* values that were statistical significant are shown in bold followed by an asterisk.

The total of malaria positive samples (n), CMC and multiclonal infection (%) by parasite and population is reported in Table 4. Overall, 130 *P*. *vivax* and 81 *P*. *falciparum* samples were genotyped (complicated and uncomplicated cases). Most of the *P*. *vivax* cases were contributed by patients from Tierralta whereas most of the *P*. *falciparum* samples were from Tumaco. This was expected due to the distinct geographic distribution of these parasites in Colombia. Among the *P*. *vivax* cases, 47.7% of the 130 samples genotyped (complicated and uncomplicated cases) had infections with more than one lineage in at least one locus. In contrast, only 14.8% out of 81 *P*. *falciparum* samples were found with multiclonal infections (Tables 2 and 3). This difference translated into a slightly higher MOI in *P*. *vivax* (1.5 vs. 1.15, Table 2) with overall more complex infections (few loci with up to three alleles).

**Table 4:** Samples included in this investigation.

	*P*. *vivax*				*P*. *falciparum*			
Population	n	Controls (MI)	CMC (MI)	Total MI	n	Controls (MI)	CMC (MI)	MI
Tierralta	63	48 (11)	15 (9)	20	3	2 (0)	1 (1)	1
Quibdó	19	11 (10)	8 (7)	17	30	21 (3)	9 (0)	3
Buenaventura	19	13 (6)	6 (4)	10	5	4 (4)	1 (0)	4
Tumaco	29	20 (9)	9 (6)	15	43	30 (2)	13 (2)	4
Total	130	92	38	62	81	57	24	12

Total of malaria samples (n), number of complicated malaria cases (CMC), and number of multiclonal infection (MI) by parasite and locality.

Overall, *P*. *vivax* loci harbored more alleles and exhibited higher heterozygosity than the loci genotyped in *P*. *falciparum*. In particular, the minimum number of alleles in *P*. *vivax* was 11 at one locus whereas in *P*. *falciparum* it was two (S2 Table). We reported measurements of genetic diversity per locus by dividing the infections in two not mutually exclusive groups. First, the genetic polymorphism per locus was calculated by considering all infections (single and multiclonal). The second group considered only infections that are monoclonal (single) or multiclonal infections at one locus only; those were the infections where multilocus genotypes could be reconstructed. This comparison showed that some alleles were only found at multiclonal infections with multiple alleles at two or more loci. The heterozygosity, however, was comparable in the two groups for the two parasites (S2 Table).

We proceeded to analyze haplotypes that could be reconstructed for both parasites by using single infections or those multiclonal infections with highly related multilocus genotypes that differed at one locus only. These analyses included 112 *P*. *vivax* samples out of the 130 and 76 of 81 *P*. *falciparum* samples. As stated earlier, complicated and uncomplicated malaria cases were matched by time of collection by randomly subsampling among uncomplicated cases that were diagnosed in an interval of up to 8 days around each complicated case. Our aim was to compare whether there were different genotypes circulating in the complicated and uncomplicated group. The number of sampled multilocus genotypes (SMG) from the human specimens, the number of distinct genotypes (G), the number of private genotypes (PG), and the Nei’s index of genetic diversity (He) estimated for each population using Haplotype Analysis software v1.04 are shown in Table 5. Overall, the mean genetic diversity was high and similar in both parasites (*Pv*-*He*: 0.969 and *Pf*-*He* = 0.822). We sampled a total of 118 private genotypes for *P*. *vivax* in terms of their geographic origin (Table 5). In contrast, out of the 76 *P*. *falciparum* samples that could be phased, the three populations shared many genotypes with only 18 private genotypes found in terms of their geographic origin.

**Table 5:** Diversity of multilocus genotypes per population estimated using Haplotype Analysis software.

	Population	Total S	Controls	CMC	Total SMG	G	PG	He
***P*. *vivax***	Tierralta	54	47	7	68	51	51	0.972
	Quibdó	14	11	3	27	18	18	0.949
	Buenaventura	15	12	3	21	19	19	0.986
	Tumaco	29	20	9	44	30	30	0.968
	Total	112	90	22	160		118	
***P*. *falciparum***	Tierralta	3	2	1	4	3	2	0.833
	Quibdó	30	21	9	33	8	6	0.780
	Tumaco	43	30	13	47	12	10	0.852
	Total	76	53	23	84		18	

Total sample size in terms of the number of blood specimens genotyped (S), estimated number of multilocus genotypes in controls and complicated malaria cases (CMC), number of sampled multilocus genotypes (SMG), number of unique genotypes (G), number of private genotypes (PG), and genetic diversity from multilocus genotypes (He) are shown by parasite for each Colombian population.

**Fig 1:**
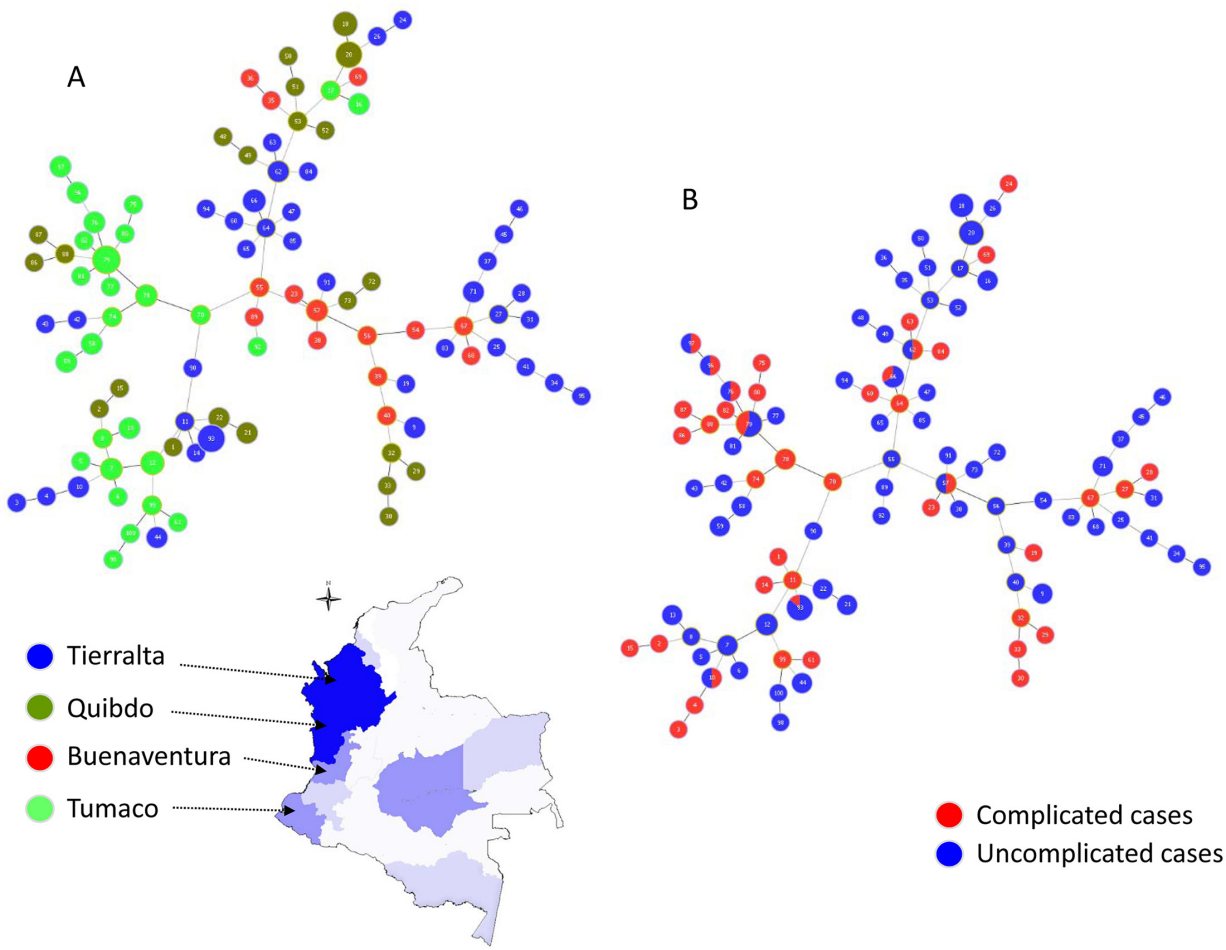
Minimum spanning tree for *P*. *vivax* constructed using goeBURST. The tree depicts the relationships among *P*. *vivax* sequence types (ST) at the nLV level (where n equals to the number of loci in our dataset: eight). Each ST is represented by a circle and the size of the circle is logarithmically proportional to the number of samples with that particular ST. The color of each circle represents the locality (A) and complicated versus uncomplicated cases (B).

A minimum spanning tree for *P*. *vivax* samples is shown in Fig 1, reflecting a genealogical relationship of 100 genotypes or sequence types (STs) at the 8LV level constructed using goeBURST with several potential putative primary founders. Each ST is represented by a circle, and the size of the circle is logarithmically proportional to the number of strains represented by the ST. The color of each circle represents the locality of the origin of the ST (Fig 1A) and complicated versus uncomplicated cases (Fig 1B). Although this was not a study on the parasite geographic structure, it is worth noting that the minimum spanning tree did not reveal a clear geographic pattern. However, some local diversification can be observed, e.g. genotypes that relate with other local genotypes (Fig 1A). Importantly, genotypes are shared between complicated and uncomplicated malaria cases showing that there is not a particular cluster of genotypes in the minimum spanning tree that could be associated with complicated cases. Indeed, some completely identical genotypes (9 out of the 100) were found in both complicated and uncomplicated malaria cases (Fig 1B). Our analyses excluded complex multiclonal infections. Given the high genetic diversity of *P*. *vivax* in these populations, our structure analyses failed to converge with this limited number of samples so we could not reliably assign isolates to *K* clusters and reveal the distribution of clusters in terms of complicated/uncomplicated malaria cases. Thus, we only reported the minimum spanning tree for *P*. *vivax* samples in this study.

**Fig 2:**
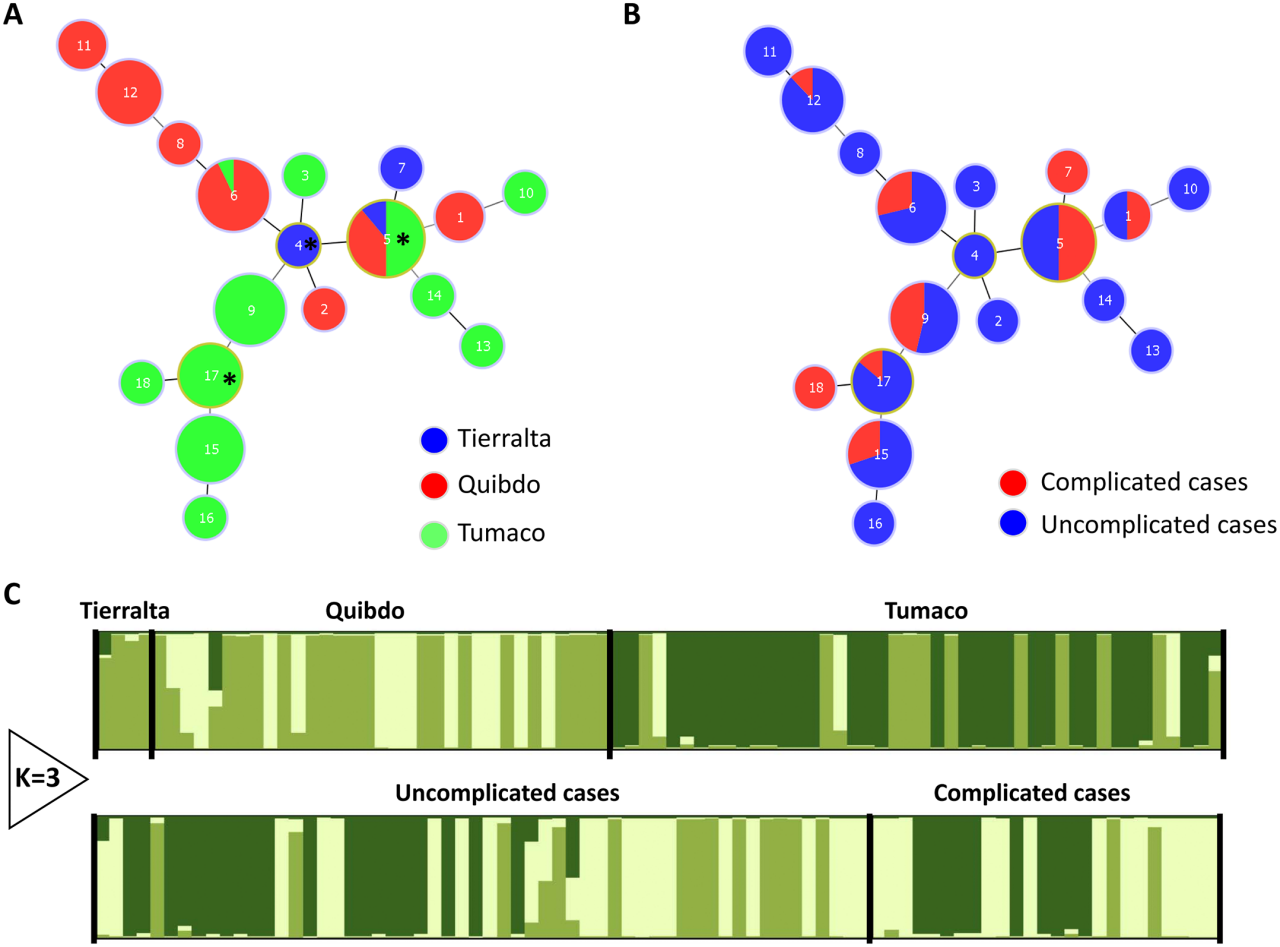
Minimum spanning tree for *P*. *falciparum* constructed using goeBURST. The tree depicts the relationships among *P*. *falciparum* sequence types (ST at the nLV level (where n equals to the number of loci in our dataset: eight). Each ST is represented by a circle and the size of the circle is logarithmically proportional to the number of samples with that particular ST. The color of each circle represents the locality (A) and complicated versus uncomplicated cases (B). Putative primary founders are indicated with an asterisk. Population structure of *P*. *falciparum* inferred from microsatellite using the STRUCTURE software (C).

In the case of *P*. *falciparum*, the 18 STs were also grouped into one clonal complex (CC) at the 8LV level by goeBURST (Fig 2) including three putative primary founders (ST4, ST5 and ST17). From these, ST5 was observed in the three populations (Tierralta, Quibdo and Tumaco) included in this analysis, whereas ST4 and ST17 were only sampled in Tierralta and Tumaco respectively (Fig 2A). Some *P*. *falciparum* genotypes (7 out of the 18) were found in both complicated and uncomplicated malaria cases (Fig 2B) and the primary founders ST5 and ST17 were also in both groups (Fig 2B). Using Structure v2.3.3, three clusters were identified for *P*. *falciparum* for the three populations (Fig 2C) and there were not specific clusters linked to complicated or uncomplicated malaria cases.

The cases infected by each parasite species were categorized into complicated and uncomplicated (see Table 1). Their infections, on the other hand, were categorized as single or multiclonal based on the set of microsatellites used in this investigation. An infection was considered multiclonal if it harbored more than one allele in at least one locus. We then explored the association between having a single or multiclonal infection with having a complicated or uncomplicated malaria event by using a Fisher exact test. The Fisher exact test yielded a significant association (*p* = 0.0035) between having a multiclonal infection and disease severity for *P*. *vivax*. In contrast, no association was observed for *P*. *falciparum* (*p* = 1.0000). Similar analyses were performed on an expanded set of samples (Table 3, n = 419 for *P*. *vivax* and n = 279 for *P*. *falciparum*) regardless of time collection. The association observed in *P*. *vivax* was also observed for this set (*p* = 0.0268) with no association for *P*. *falciparum* (*p* = 0. 432). The pattern in *P*. *vivax* persisted even when we considered as multiclonal infections only those having multiple alleles in two loci or more. The 2x2 contingency tables for both parasites are given in Table 3. The pattern in *P*. *vivax* cannot be explained by differences on the average parasitemia that affected our capacity to detect lineages. No differences in parasitemia were observed between the complicated and the non-complicated malaria groups (p = 0.712, Mann Whitney test on medians).

## Discussion

There have been multiple epidemiological investigations aiming to explore the relationship between MOI and/or the frequency of multiclonal infections with variables of epidemiological interest, including but not limited to clinical endpoints. Examples of such studies are shown in S1 Table. The variation of the genetic markers used and the broad spectrum of epidemiological variables investigated hampered our ability to compare findings across studies. Nevertheless, there were some emerging patterns. For example, in the handful of studies where *P*. *vivax* was compared with *P*. *falciparum*, patients with *P*. *vivax* malaria harbored multiclonal infections more often than those with *P*. *falciparum* malaria [8, 11, 13, 45, 46](S1 Table). Our findings are consistent with this global trend. The observed higher frequency of multiclonal infections in *P*. *vivax* could be the result of hypnozoites accumulating in the liver yielding multiple relapses of distinct genotypes. If this were the only factor, it would imply that patients received incomplete treatment with primaquine, a drug that is prescribed to treat uncomplicated *P*. *vivax* malaria in Colombia and other Latin-American countries. Our observation, however, cannot be taken as evidence of lack of compliance with the local drug policy. It is possible that *P*. *vivax* patients remained asymptomatic for a long period of time [47] facilitating superinfections because antimalarial treatment was not provided. A factor that could also contribute to this pattern is that *P*. *vivax* has higher prevalence and genetic diversity in this region when compared to *P*. *falciparum*; thus, ecological differences in terms of transmission are easier to detect and could partially explain the higher frequency of multiclonal infections as a result of coinfections or superinfections [29, 30]. The differential contribution of these and other factors to the observed high frequency of multiclonal infections in *P*. *vivax* is a matter that requires additional investigations.

Many studies indicate that MOI is better explained by exposure as it correlates with age [48–51]; these investigations have been carried out mostly in areas with higher transmission than the one surveyed in this study. It may be possible that superinfections are more likely if the patients are subclinical for long periods of time due to acquired immunity; thus the frequency of multiclonal infections is expected to correlate with age. Because our study design focused on contrasting complicated with uncomplicated cases in several localities, it did not allow us to properly test a relationship between age and the frequency of multiclonal infections.

In the context of disease severity, controlled epidemiologic investigations have found that the frequency of multiclonal *P*. *falciparum* infections is not associated with clinical symptoms or severity of malaria cases. In particular, groups of severe and mild malaria cases have been compared independently in The Gambia [22], Senegal [52], Gabon [23], Côte d'Ivoire [12], and Thailand [53] with each study showing that the numbers of genotypes per infection were similar between groups. These observations are consistent with our findings in *P*. *falciparum* with the caveat that we only had a few complicated malaria cases in this low transmission setting.

Contrary to the *P*. *falciparum* pattern, we found that having a multiclonal infection is associated with disease severity in *P*. *vivax*. Our observations on *P*. *vivax* are consistent with those found in experimental infections using rodent malaria models [5, 16, 17] where multiclonal infections correlated with disease severity. At this point it is worth noting that in some rodent malaria models (*Plasmodium chabaudi*), multiclonal infections may lead to an increase on the average virulence at the population level since natural selection will favor highly competitive parasites [5, 16]. However, in other rodent malaria model (*Plasmodium yoelii*) this was not observed [17]. Thus, having an association between multiclonal infections and disease severity does not necessarily indicate a selective advantage for more virulent parasites in that population. Consistent with this scenario, we found no evidence indicating that there were particular parasites being selected toward “higher virulence”.

In particular, our haplotype networks did not detect differences in the genotypes circulating between complicated and uncomplicated cases in the two malarial parasites. This suggests that any effect on disease severity may be due to differences in the host (e.g. differences in acquired immunity) or the actual composition of the infection (multiclonal versus single infections) rather than the genetic makeup of the circulating parasites. A limitation of the haplotype network analyses as a proxy of the parasites genealogies, however, is that by excluding complex multiclonal infections we did not consider some specific genotypes in both groups of cases (complicated and uncomplicated). Importantly, the loci sampled were not linked to any known virulent factor. A more comprehensive analysis that incorporates the parasites genealogies will require bigger samples sizes in terms of the number of complicated malaria cases and the use of approaches such as genotyping by sequencing.

Although differences in disease severity between *P*. *vivax* and *P*. *falciparum* are expected given their distinct biological characteristics, the observed association in *P*. *vivax* may also provide insights on the limitations that simple measurements such as MOI or the frequency of multiclonal infections may have when testing in the field predictions regarding disease severity derived from experimental models. Experimental models control for specific variables because they aim to test evolutionary hypotheses; such controls are not possible to implement or are not considered in field settings. As an example, multiclonal infections driven by genetically related parasite lineages (siblings) are expected to reduce virulence in the population [54–56]. This prediction is consistent with the observation that many *P*. *falciparum* multiclonal infections were highly related (siblings) since they had multiple alleles at one locus only. Unfortunately, the limited number of complicated cases in our field sites hampered our ability of performing any meaningful tests. Notably, the association between multiclonal infections and disease severity in *P*. *vivax* holds even when we changed our definition of multiclonal infections to one requiring more than one loci with multiple alleles. At this point, it seems that a major noticeable difference between the infections caused by the two parasites is that *P*. *vivax* multiclonal infections have lineages that were more distantly related among them (e.g. more loci with multiple alleles) than in the case of the *P*. *falciparum*.

It is also worth noting that the association found in *P*. *vivax* may reflect host differences rather than being a consequence of having multiple parasite lineages competing during the course of an infection. A possibility worth exploring is that malaria exposure is expected to be relatively low in this population when compared with areas of high transmission where patients may have developed acquired immunity. Under this “low levels of acquired immunity scenario”, multiclonal infections may present a major challenge for the patient leading to more severe clinical disease manifestations. If immunity were the factor modulating the clinical outcomes of multiclonal infections in *P*. *vivax*, the association between multiclonal infections and disease severity may be found in areas with low transmission more often than in areas with high transmission. The importance of exposure and immunity can be inferred from studies carried out in areas of high transmission where the frequency of multiclonal infections and MOI could be better explained by age [48–51]. Importantly, having a suitable independent metric for acquired immunity/exposure (other than age) is essential for testing this hypothesis. Consistent with this scenario of low acquired immunity, preliminary analysis of those individuals infected with *P*. *vivax* shown that most of them displayed very low anti-parasite antibody titers against *P*. *vivax* circumsporozoite protein (*Pv*CS; median 0.99, IQR 0.8–1.3) and the merozoite surface protein-1 (*Pv*MSP-1; median 0.95, IQR 0.8–1.2) expressed as arbitrary units with values > 1.0 considered as positive. Moreover, no significant differences in the frequency of responders were observed between single and multiclonal infection in both control and complicated patients (p>0.05). These results are consistent with previous studies carried out in low transmission areas [57, 58]. Having comparable genetic and immunologic data from low and high transmission areas would be essential to further understand the relationship between multiclonal infections and clinical outcomes in *P*. *vivax*.

In summary, this study provides additional evidence for epidemiological differences between *P*. *vivax* and *P*. *falciparum* malarias in low transmission settings. We found that *P*. *vivax* infections were more diverse than *P*. *falciparum* infections even in these settings. Regardless of the small sample size, we found a significant association between multiclonal infections and disease severity in *P*. *vivax*; a pattern that is consistent with previous observations made in rodent malaria models. This association was not found in *P*. *falciparum*. The contrasting pattern between *P*. *vivax* and *P*. *falciparum* could be explained, at least in part, by the fact that *P*. *vivax* infections had lineages that were more distantly related among them than in the case of the *P*. *falciparum* multiclonal infections. The low numbers of complicated malaria cases for both parasites is a limitation in this study; thus, additional efforts should be made in order to better characterize how clinical outcomes may be affected by multiclonal infections in low transmission areas. Comparisons across *P*. *vivax* endemic areas will allow exploring the epidemiological and ecological contexts where multiclonal infections may be associated with disease severity.

## Supporting Information

S1 Table Studies reporting multiclonal infections in *P*. *vivax* and *P*. *falciparum*. (XLSX)

S2 Table Genetic polymorphism found in *P*. *vivax* and *P*. *falciparum* samples. Fragment size ranges per locus, number of alleles per locus, and heterozygosity (He) per locus are shown.
(DOCX)
