# Supplementary material for: Multiplicity of Infection and Disease Severity in Plasmodium vivax
Source: PLoS Negl Trop Dis. 2016 Jan 11;10(1):e0004355. doi: 10.1371/journal.pntd.0004355 (PMC4709143; doi:10.1371/journal.pntd.0004355)
Supplement: S2 Table — Fragment size ranges per locus, number of alleles per locus, and heterozygosity (He) per locus are shown. (DOCX) [file pntd.0004355.s002.docx]

**S2 Table. Genetic polymorphism found in *P. vivax* and *P. falciparum* samples.**

| **Locus** | **Allele Ranges^a^** | **No. of Alleles^a^** | **He^a^** | **Allele Ranges^b^** | **No. of Alleles^b^** | **He^b^** |
| --- | --- | --- | --- | --- | --- | --- |
| ***P. vivax* (n=130)** |  |  |  |  | | |
| **MS2** | 181-275 | 20 | 0.844 | 181-241 | 18 | 0.828 |
| **MS5** | 163-205 | 11 | 0.856 | 163-202 | 10 | 0.855 |
| **MS6** | 212-254 | 14 | 0.77 | 212-252 | 12 | 0.767 |
| **MS15** | 235-307 | 18 | 0.836 | 235-301 | 14 | 0.821 |
| **14.185** | 242-284 | 16 | 0.778 | 248-284 | 12 | 0.749 |
| **8.332** | 216-261 | 14 | 0.656 | 216-261 | 13 | 0.656 |
| **2.21** | 83-127 | 12 | 0.708 | 83-127 | 12 | 0.719 |
| **10.29** | 110-148 | 14 | 0.879 | 110-148 | 14 | 0.88 |
| **3.35** | 113-157 | 16 | 0.912 | 113-157 | 16 | 0.912 |
|  |  |  |  |  |  |  |
| **Total (all loci)** |  |  | **0.804** |  |  | **0.799** |
| ***P. falciparum* (n=81)** | |  |  |  | | |
| **POLYa** | 150-180 | 9 | 0.622 | 150-180 | 5 | 0.56 |
| **TAA60** | 69-97 | 6 | 0.619 | 69-85 | 5 | 0.574 |
| **ARA2** | 58-74 | 6 | 0.599 | 58-74 | 5 | 0.545 |
| **Pfg377** | 92-172 | 5 | 0.491 | 94-172 | 4 | 0.479 |
| **TAA109** | 136-202 | 4 | 0.16 | 136-202 | 3 | 0.069 |
| **TAA81** | 115-127 | 4 | 0.67 | 115-127 | 3 | 0.658 |
| **TAA42-3** | 185-187 | 2 | 0.355 | 185-187 | 2 | 0.375 |
| **TA40** | 160-162 | 2 | 0.022 | 160-162 | 2 | 0.024 |
| **PfPK2** | 161-173 | 5 | 0.567 | 161-173 | 4 | 0.562 |
|  |  |  |  |  |  |  |
| **Total (all loci)** |  |  | **0.456** |  |  | **0.427** |

**^a^**Considers all samples including those where multilocus genotypes could not be phased;

**^b^**excludes complex infections where multilocus genotypes could not be inferred (see text).

Fragment size ranges per locus, number of alleles per locus, and heterozygosity (He) per locus are shown.
